# Temperature has a major effect on the cuticular wax composition of bilberry (*Vaccinium myrtillus* L.) fruit

**DOI:** 10.3389/fpls.2022.980427

**Published:** 2022-09-20

**Authors:** Priyanka Trivedi, Linards Klavins, Anne Linn Hykkerud, Jorens Kviesis, Didzis Elferts, Inger Martinussen, Maris Klavins, Katja Karppinen, Hely Häggman, Laura Jaakola

**Affiliations:** ^1^Department of Ecology and Genetics, University of Oulu, Oulu, Finland; ^2^Department of Environmental Science, University of Latvia, Riga, Latvia; ^3^NIBIO, Norwegian Institute of Bioeconomy Research, Ås, Norway; ^4^Faculty of Biology, University of Latvia, Riga, Latvia; ^5^Department of Arctic and Marine Biology, UiT The Arctic University of Norway, Tromsø, Norway

**Keywords:** cuticular wax, berry, temperature, latitudinal gradient, triterpenoids, fatty acids, phytotron

## Abstract

Cuticle is the first layer protecting plants against external biotic and abiotic factors and is responsive to climatic factors as well as determined by genetic adaptations. In this study, the chemical composition of bilberry fruit cuticular wax was investigated through a latitudinal gradient from Latvia (56°N 24°E) through Finland (65°N 25°E) to northern Norway (69°N 18°E) in two seasons 2018 and 2019. Changes in the major cuticular wax compounds, including triterpenoids, fatty acids, alkanes, aldehydes, ketones, and primary alcohols, were detected by GC-MS analysis. Generally, a decreasing trend in the proportion of triterpenoids from southern to northern latitudes, accompanied with an increase in proportion of fatty acids, aldehydes, and alkanes, in bilberry fruit cuticular wax was observed. A correlation analysis between climatic factors with proportion of wax compounds indicated that temperature was the main factor affecting the cuticular wax composition in bilberries. A controlled phytotron experiment with southern and northern bilberry ecotypes confirmed the major effect of temperature on bilberry fruit cuticular wax load and composition. Elevated temperature increased wax load most in berries of northern ecotypes. The level of triterpenoids was higher, while levels of fatty acids and alkanes were lower, in wax of bilberry fruits ripened at 18°C compared to 12°C in both northern and southern ecotypes. Based on our results, it can be postulated that the predicted increase in temperature due to climate change leads to alterations in fruit cuticular wax load and composition. In northern ecotypes, the alterations were especially evident.

## Introduction

All aerial plant parts, including fruits, are covered with a protective cuticle. This is the first barrier to protect plants against pathogens and UV-B radiation in addition to preventing non-stomatal water loss (Yeats and Rose, 2013). The plant cuticle is composed of a polyester called cutin and cuticular wax. The cuticular wax is present as intracuticular wax, an amorphous mixture of lipids embedded in the cutin, and outermost epicuticular wax overlaying cuticle (Barthlott et al., 2017). The physiochemical properties of cuticular wax mediate the plants’ responses to various biotic and abiotic stresses and are associated with plant adaptability (Dodd and Poveda, 2003). Cuticular wax is composed of very long chain (VLC) fatty acids and their derivatives, such as alkanes, aldehydes, ketones, and alcohols as well as secondary metabolites including triterpenoids. The VLC fatty acid compounds accumulate from the wax biosynthetic pathway in epidermal cells, while triterpenoids are synthesized through a separate mevalonate pathway of the epidermal cells (Kunst and Samuels, 2003; Yeats and Rose, 2013).

Stress modeling in addition to field and controlled studies have revealed that plants adjust their cuticular wax load and composition to adapt to changing environment and abiotic stress conditions, such as drought, temperature fluctuations, and radiation level (Shepherd and Griffiths, 2006; Guo et al., 2015, 2021; Gao et al., 2016). Drought stress has been shown to increase cuticular wax load and the content of alkanes in Arabidopsis (Kim et al., 2007; Kosma et al., 2009), while enhanced UV-B radiation was shown to increase the content of alkanes and primary alcohols in cucumber (*Cucumis sativus*) cotyledon (Fukuda et al., 2008). In addition, it was reported that the average carbon chain length of alkanes in leaf wax increased with the increasing temperature through a temperature gradient (Wang et al., 2018). However, the knowledge on the effect of temperature on cuticular wax profile, specifically the triterpenoid fraction, is still scarce. Moreover, among fruits and berries, the reports on the effect of temperature on cuticular wax have mostly been limited to post-harvest storage studies (Wu X. et al., 2017; Chu et al., 2018; Trivedi et al., 2019b).

Wild bilberry (*Vaccinium myrtillus* L.), also known as the European blueberry, is distributed widely across Europe and Northern Asia (Nestby et al., 2011). Bilberry is one of the economically most important berry species of Northern Europe and valued for its nutraceutical and health-beneficial properties (Kangas and Markkanen, 2001; Jimenez-Garcia et al., 2013). The development and ripening of bilberry fruit is regulated by genetic and environmental factors, which both are affected by the geographical growth location (Åkerström et al., 2010; Zoratti et al., 2015). In our previous study, we analyzed the cuticular wax composition of bilberry fruit (ecotype from Oulu, Finland) in various developmental stages (Trivedi et al., 2021). It was observed that cuticular wax biosynthesis proceeds during the fruit ripening and is accompanied by wax accumulation.

The global annual mean temperature has increased by 1.1^°^C since 1850–1900 and is predicted to increase 2.5–4^°^C by the end of this century (IPCC Sixth Assessment Report, 2022). Therefore, it is imperative to study the effect of increase in temperature on the composition of cuticular waxes on fruit of economically important plants such as bilberry. Since cuticular wax load and composition are important for fruit quality and plant adaptations, their response to temperature is of importance both from a commercial and an ecological perspective. Latitudinal gradient represents variation in environmental conditions, such as temperature and precipitation, and can be used to study the effect of environment on plant cuticular wax traits. Studying the response of cuticular wax to climatic variations through a latitudinal gradient may be useful in assessing how berry plants may respond to climate change. The aim of the present study was to investigate the effect of temperature and precipitation on the composition of cuticular wax of bilberry fruit through a latitudinal gradient from southern location in Latvia through Finland to the northern Norway. In addition, a controlled experiment in phytotrons was performed to study the effect of temperature on the cuticular wax composition and quantity in bilberry fruit.

## Materials and methods

### Plant material and phytotron experiment

Ripe bilberry fruits grown in natural habitats were collected by using forceps from three latitudes, including southernmost latitude Latvia (56°N 24°E), Finland (65°N 25°E), and northern Norway (69°N 18°E) in consecutive years 2018 and 2019. Samples were collected from the same locations within a radius of 10 m in both the years. Uniform sized berries at the same ripening stage were harvested for wax extraction from five different sub-locations and in triplicates from each sub-location (Supplementary Table 1).

For phytotron experiments, bilberry plants with large green unripe berries were collected from forests in Alnarp, Sweden (southern clones, 55°N 13°E) and Tromsø, Norway (northern clones, 69°N 18°E) in the summer season of 2019. The plant shoots were kept in water in two controlled growth chambers (phytotrons), at temperatures of 12 and 18°C in each, for 14 days until the berries reached ripeness. The humidity was adjusted to 70%, photoperiod was 21 h light/3 h dark, and light intensity was adjusted to 100 μmol m^–2^ s^–1^. The berries were collected for wax extraction in triplicates by forceps.

### Cuticular wax extraction and determination of wax amount

The cuticular wax of ripe bilberry fruits was extracted immediately after collection at ambient temperature by dipping one hundred berries in 15 ml of chloroform (Sigma-Aldrich, St. Louis, MO, United States) for 1 min. The chloroform extract was evaporated to dryness under flow of nitrogen (AGA, Guildford, United Kingdom) at room temperature and the dry weight was measured. The cuticular wax amount was expressed as mg per berry.

### Preparation of samples for GC-MS

Determination of fatty acids in the cuticular wax extracts was carried out by methylation using 14% BF_3_ methanol solution, before the GC-MS analysis according to Duron and Nowotny (1963) with minor modifications. Briefly, after the initial addition of internal standard of methyl heptadecanoate, 0.5 ml toluene and 3 ml of 14% BF_3_ were added to about 20 mg of wax sample, agitated, and kept at 60–65°C for 180 min. After cooling, extraction of lipids was conducted using hexane/diethyl ether (1:1, 3 mL) and 5% w/v aqueous sodium chloride (5 mL) was added as described earlier by Dobson et al. (2012). The organic layer was then separated by centrifugation and the solvents were evaporated until dryness over anhydrous Na_2_SO_4_. After redissolution in hexane, the samples were filtered through a 0.45 μm PTFE membranefilter (Cole-Parmer, IL, United States) and analyzed by GC-MS. Methylation reagent provided methylation of carboxylic acid groups and left the hydroxyl groups intact for later silylation. To identify the compounds containing –OH groups the sample was derivatized using BSTFA after methylation by dissolving it in 1 mL pyridine and 50 μL of BSTFA. The samples were heated at 75^°^C for 30 min and cooled before GC-MS analysis.

### GC-MS analysis

GC-MS analysis was performed as described previously in Trivedi et al. (2021). The chromatographic separation of FAMEs (fatty acid methyl esters) was done using Omegawax 250 column (30 m × 0.25 mm, 0.25 μm; Supelco, Bellefonte, PA, United States) with a poly (ethylene glycol)-based stationary phase with PerkinElmer Clarus 580 system equipped with a Clarus SQ 8C mass-selective detector (Waltham, MA, United States). Determination of the other chemical components was performed by Elite-5MS column (30 m × 0.25 mm, 0.25 μm; PerkinElmer, United States) as TMS (trimethylsilyl) derivatives. GC temperature program was set at initial 75^°^C and held for 2 min, the temperature was increased to 150^°^C at a rate of 20^°^C min^–1^ and then further increased to 310^°^C at a rate of 4^°^C min^–1^, after reaching 310^°^C a final isothermal step was held for 5 min. Injection port and interface were set at 290^°^C and injection volume was 1 μL. Helium was used as the carrier gas with the total flow rate 1.0 mL min^–1^ in split mode (1:10) with the flow of 10.0 mL min^–1^. Electron impact voltage was set to 70 eV with the scan range from 35 to 750 m/z. The quantification of chemical components present in the cuticular wax samples was determined by constructing calibration curves (1.5–500 μg mL^–1^) and relating the peak area of represent compounds to concentration of the reference compound. Methylheptadecanoate, ursolic acid, 1-dodecanal, (±)-α-tocopherol, 1-octadecanol, and n-tetracosane were used as a set of reference compounds to represent chemical classes of fatty acids, triterpenoids, alkyl-aldehydes (ketones), tocopherols, alkanols, and alkanes, respectively. System control, data acquisition, analysis, and processing were done by TurboMass Ver6.0.0 user interface with NIST MS 2.2 Library (FairCom Corp., United States). The results have been expressed as μg of quantified substance per mg of berry cuticular wax.

### Meteorological data

The weather data was obtained from the closest weather stations (50 km radius) from the bilberry collection area. The meteorological data was collected from weather stations in Oulu (Finland) by VTT Technical Research Centre of Finland, in Tromsø (Norway) by Norwegian Institute of Bioeconomy (NIBIO) and in Riga (Latvia) by Latvian Environment, Geology and Meteorology Centre. The collected data included: average temperature of 8 weeks before harvest in °C (T_avg_), maximum temperature of 8 weeks before harvest in °C (T_max_), minimum temperature of 8 weeks before harvest in °C (T_min_), and average of precipitation of 8 weeks before harvest in mm (P_avg_) (Supplementary Table 2).

### Statistical analysis

The relationship between the cuticular wax composition and environmental factors was analyzed using redundancy analysis (RDA) as implemented in R 1.3.1093 (R Core Team, 2020) packages vegan (Oksanen et al., 2019). The dependent matrix included cuticular wax components (ketones, aldehydes, alkanes, fatty acids, triterpenoids) and the constraining matrix included climatic variables (T_min_, T_avg_, T_max_, and P_avg_). RDA was done separately for years 2018 and 2019 and for the combined data of the 2 years. Significance of RDA was analyzed using ANOVA including permutation test for RDA. Before performing the RDA analysis, a stepwise regression analysis was run in Minitab to select the climatic factors (T_min_, T_avg_, T_max_, and P_avg_) prior to harvest to find the most affecting factors and time-periods variating in wax composition in bilberry.

A mixed-effect model was performed to study the variation in wax load, where T_min_, T_avg_, T_max_, and P_avg_ and location were fixed factors, while year and sublocation were random factors. Significant differences (*p* < 0.05) in wax load and concentration of wax compounds in phytotron experiment were analyzed by independent sample *t*-test using SPSS Statistic program version 26.0 (IBM, Chicago, United States). One-way analysis of variance (ANOVA; *p* < 0.05) was performed for analyses of significant differences in wax compounds for the three latitudes using the SPSS program.

## Results

### Berry cuticular wax load through the latitudinal gradient

**FIGURE 1:**
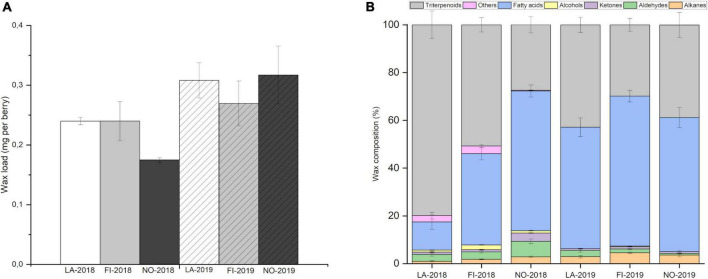
Cuticular wax load and profile through latitudinal gradient. **(A)** Amount of cuticular wax (mg per berry) in bilberry fruits from Latvia (LA), Finland (FI), and Norway (NO) in years 2018 and 2019. The data is shown as mean ± SD of five replicates. **(B)** Cuticular wax profile in bilberry fruit through a latitudinal gradient from LA through FI and NO in 2018 and 2019. Error bars indicate Standard Error (*n* = 5).

Cuticular wax load was studied on berries sampled from their natural habitats through the latitudinal gradient in the summer seasons of 2018 and 2019. A variation was observed in the cuticular wax load between the studied latitudes and between the two seasons (Figure 1A). The results from 2018 showed lower wax load in Northern Norway compared to the other two locations in Latvia and Finland. In 2019, a higher bilberry fruit wax load was generally observed in all the three studied latitudes compared to season 2018 (Figure 1A). Berries grown in Latvia and northern Norway in 2019 had slightly higher wax load than the location in Finland. Correlation analysis on the total wax load with meteorological data indicate a strong negative correlation between the total wax load and the precipitation in the second month before ripening (Supplementary Table 3). Analyses by mixed-effect model indicated that the growth location significantly affected the variation of total wax amount in bilberry fruit (*p* < 0.0001) (Supplementary Table 4).

### Composition of bilberry cuticular wax through the latitudinal gradient

The major chemical compounds of bilberry fruit cuticular wax found in all three locations were triterpenoids, fatty acids, alkanes, aldehydes, ketones, and primary alcohols (Figure 1B). Secondary alcohols or esters were not detected. Differences in concentrations and relative proportions of wax compounds were found in bilberry cuticular wax through the latitudinal gradient and between years (Figure 1B). In 2018, a clear trend of decrease in proportion of triterpenoids in berry wax was observed from southern latitudes in Latvia (79.9% triterpenoids of total wax), through location in Finland (50.7% triterpenoids of total wax) to northern Norway (27.4% triterpenoids of total wax). A simultaneous trend of increase in proportion of fatty acids and alkanes was observed from southern to northern latitudes (Figure 1B). In 2018 season, triterpenoids were the dominant compounds in bilberry fruit cuticular wax in Latvia and Finland locations, while fatty acids dominated in northern Norway (Figure 1B). In 2019 season, a similar trend of decrease in triterpenoid proportions from southernmost location Latvia (42.9% triterpenoids of total wax) to the location in Finland (29.8% triterpenoids of total wax) was observed along with increase in fatty acid and alkane proportions (Figure 1B). However, in the samples from northern Norway, the same trend in triterpenoid and fatty acid proportions was not observed in 2019. Fatty acids were the dominant compounds in all locations followed by triterpenoids in 2019 (Figure 1B).

### Profile of triterpenoids and very long chain compounds in cuticular wax of bilberry fruit

The triterpenoid profile and the dominant triterpenoid compound varied between the geographical locations through the latitudinal gradient. Oleanolic acid was the dominant triterpenoid in both 2018 and 2019 seasons in bilberry fruit cuticular wax of Latvian location (Table 1). It constituted 54 and 43% of triterpenoids in 2018 and 2019 season, respectively. From total wax load, oleanolic acid made up 43 and 7% in 2018 and 2019 season, respectively, in Latvian location. Instead, the cuticular wax in bilberry fruit from northern Norway and Finland showed β-amyrin as the dominant triterpenoid in both years (Table 1). In Finnish berries, β-amyrin constituted 38% of triterpenoids and 19% of total wax in 2018, while in 2019, it constituted 21% of triterpenoids and 7% of total wax. In the northern Norwegian berries, β-amyrin made 25% of terpenoids and 7.5% of total wax content in 2018 and 25 and 10% in 2019 season. Ursolic acid was found in higher quantity in Latvian samples, especially in season 2018 (29% of terpenoids) when compared to the Finnish and Norwegian samples. Lupeol was found in higher quantity in the Finnish wax samples (10% of triterpenoids in year 2018, and 19% in 2019) as compared to Latvian and Norwegian berries.

**TABLE 1:** Quantities (μg/mg of berry cuticular wax) of triterpenoids in ripe bilberry wax through a latitudinal gradient.

Cuticular wax compounds	2018			2019		
		
	Latvia	Finland	Norway	Latvia	Finland	Norway
**Triterpenoids**						
Friedelin	8.02 ± 5.24	nd	18.56 ± 12.23	8.38 ± 1.80	18.34 ± 10.27	16.37 ± 12.09
Oleanolic acid	372.13 ± 92.23*	112.76 ± 67.32*	11.86 ± 4.44*	22.4 ± 11. 5*	27.17 ± 8.61*	4.13 ± 9.23*
Ursolic acid	204.04 ± 69.21*	47.73 ± 31.52*	11.29 ± 7.43*	8.87 ± 1.27	2.65 ± 5.93	nd
β-Amyrin	53.15 ± 18.06*	168.03 ± 56.33*	22.46 ± 9.30*	16.67 ± 6.44	31.24 ± 14.65	33.22 ± 12.19
α-Amyrin	26.64 ± 15.71*	40.33 ± 8.05*	11.60 ± 4.38*	11.39 ± 2.52*	27.04 ± 11.54*	27.37 ± 8.76*
Lupeol	11.07 ± 7.22*	45.17 ± 8.23*	2.35 ± 5.25*	9.97 ± 2.43*	28.06 ± 12.97*	24.63 ± 6.16*
Olean-2.12-dien-28-oic acid 3-one	nd	nd	nd	9.35 ± 2.76	6.13 ± 6.47	17.20 ± 9.78
Ursa-2.12-dien-28-oic acid	nd	nd	nd	17.71 ± 8.38	5.46 ± 7.48	6.03 ± 13.49
Methylenecycloartanol	nd	nd	nd	10.18 ± 3.81	nd	nd
γ-Taraxasterol	4.44 ± 3.41	24.64 ± 4.50	nd	13.69 ± 6.82	nd	nd
Bauerenol	nd	nd	nd	16.25 ± 3.97	nd	nd
Uvaol	4.03 ± 1.64	9.79 ± 11.08	10.04 ± 5.27	nd	nd	nd
Total	683.52	439.92	88.15	144.92	146.10	128.95

Data is means ± SD of five replicates (sub-locations).

*Indicates statistically significant differences between locations means (P < 0.05).

Unlike triterpenoids, the dominant component of fatty acids, alkanes and ketones remained same through latitudinal gradient. Montanic acid was the dominant fatty acid constituting 50, 41, and 30% of fatty acids (5.8, 15.9, and 17.9% of the total wax) in Latvian, Finnish, and Norwegian wax samples, respectively, in 2018 (Table 2). In 2019, its contribution to the total fatty acids wax 39, 36, and 34% (20, 23, and 19.9% of total wax) in Latvian, Finnish, and Norwegian berries, respectively. 2-heneicosanone was the dominant ketone, while heptacosane was the dominant alkane in all the studied berry wax samples. Octacosanal was the dominant aldehyde in the 2018 samples of Latvia, Finland, and Norway, as well as in the samples of Norway and Latvia in 2019 (Table 2).

**TABLE 2:** Quantities (μg/mg of berry cuticular wax) of very long chain aliphatic compounds in ripe bilberry wax through a latitudinal gradient.

Cuticular wax compounds	2018			2019		
		
	Latvia	Finland	Norway	Latvia	Finland	Norway
**Fatty acids**						
Oleic acid	0.23 ± 0.05*	0.62 ± 0.05 *	0.59 ± 0.21*	nd	nd	nd
Stearic acid	1.02 ± 0.13*	2.86 ± 0.47*	1.88 ± 0.35*	0.82 ± 0.31*	1.69 ± 0.26*	1.53 ± 0.76*
Nonadecanoic acid	nd	1.11 ± 0.55	0.55 ± 0.33	0.45 ± 0.14*	0.61 ± 0.11*	1.38 ± 0.81*
Arachidic acid	1.87 ± 0.67*	41.48 ± 6.83*	40.42 ± 8.17*	12.89 ± 5.56	22.62 ± 5.30	22.15 ± 12.94
Behenic acid	1.48 ± 0.51*	3.91 ± 0.37*	4.09 ± 0.95*	1.80 ± 0.79*	5.04 ± 0.66*	4.11 ± 1.45*
Lignoceric acid	2.66 ± 0.71*	6.13 ± 1.04*	9.61 ± 1.97*	5.52 ± 2.28	9.98 ± 1.13	11.06 ± 5.79
Hyenic acid	0.42 ± 0.15*	2.19 ± 0.60*	1.49 ± 0.49*	nd	2.14 ± 0.39	2.69 ± 1.84
Cerotic acid	21.25 ± 3.62*	60.54 ± 7.50*	53.06 ± 10.91	46.30 ± 22.9	63.59 ± 3.17	55.46 ± 9.52
Carboceric acid	1.13 ± 0.36*	4.73 ± 1.39*	2.70 ± 0.52*	nd	nd	nd
Montanic acid	38.60 ± 6.03*	134.55 ± 13.32*	57.65 ± 7.29*	61.35 ± 35.94	83.35 ± 14.58	64.79 ± 11.88
Nonacosanoic acid	0.94 ± 0.35	2.24 ± 0.69	1.09 ± 0.16	1.11 ± 0.38	2.33 ± 0.47	3.03 ± 1.89
Melissic acid	nd	53.02 ± 12.80	nd	nd	27.72 ± 17.35	13.42 ± 3.52
Total	69.79	313.37	184.50	148.42	226.0	185.98
**Ketones**						
2-Nonanone	nd	nd	nd	nd	nd	nd
2-Undecanone	nd	nd	0.15 ± 0.05	nd	nd	nd
2-Tridecanone	1.79 ± 0.22*	1.50 ± 0.83*	0.20 ± 0.15*	1.37 ± 0.81	1.12 ± 0.64	nd
2-Nonadecanone	0.42 ± 0.02*	0.30 ± 0.04*	0.25 ± 0.04*	0.11 ± 0.03*	0.18 ± 0.02*	0.12 ± 0.06*
2-Heneicosanone	3.49 ± 0.55*	6.14 ± 1.80*	10.47 ± 2.63*	1.60 ± 1.30	2.87 ± 0.92	3.15 ± 1.58
2-Docosanone	nd	nd	0.12 ± 0.07	nd	nd	nd
Total	5.34	7.96	11.30	3.07	3.72	1.74
**Aldehydes**						
Octadecanal	nd	0.25 ± 0.12	0.06 ± 0.02	nd	nd	nd
Tetracosanal	0.41 ± 0.04*	0.11 ± 0.16*	0.44 ± 0.18*	0.18 ± 0.09	0.03 ± 0.07	nd
Pentacosanal	0.41 ± 0.01	0.34 ± 0.11	0.53 ± 0.34	0.26 ± 0.16	0.22 ± 0.19	nd
Hexacosanal	3.92 ± 1.40*	4.05 ± 2.60*	8.12 ± 2.71*	2.39 ± 1.44	0.77 ± 0.32	nd
Heptacosanal	0.48 ± 0.06*	1.21 ± 0.34*	0.62 ± 0.15*	0.45 ± 0.35	0.92 ± 0.63	0.41 ± 0.32
Octacosanal	9.10 ± 3.19	14.13 ± 7.07	9.54 ± 4.15	3.44 ± 2.12*	1.33 ± 0.36*	0.69 ± 0.38*
Triacontanal	4.15 ± 1.71	4.27 ± 2.58	1.75 ± 1.44	nd	1.76 ± 1.54	nd
Hentriacontanal	nd	nd	nd	nd	0.56 ± 0.92	nd
Dotriacontanal	nd	nd	nd	nd	0.74 ± 1.24	nd
Total	18.46	26.77	21.10	7.01	6.34	1.72
**Alkanes**						
Tetracosane	0.30 ± 0.12*	0.61 ± 0.23*	0.46 ± 0.07*	0.17 ± 0.04*	0.59 ± 0.13*	0.67 ± 0.23*
Pentacosane	1.16 ± 0.23*	3.02 ± 0.84*	2.15 ± 0.61*	0.96 ± 0.35*	2.82 ± 1.60*	2.44 ± 0.86*
Hexacosane	0.36 ± 0.07*	0.78 ± 0.19*	0.51 ± 0.07*	0.25 ± 0.09*	0.83 ± 0.07*	0.74 ± 0.28*
Heptacosane	2.13 ± 0.54*	6.76 ± 1.80*	3.79 ± 1.16*	2.48 ± 0.95*	6.12 ± 1.46*	3.60 ± 0.23*
Octacosane	0.26 ± 0.15	nd	0.41 ± 0.20	0.65 ± 0.56	1.24 ± 1.85	0.50 ± 0.18
Nonacosane	1.33 ± 0.58	3.77 ± 1.24	nd	1.25 ± 0.49	2.31 ± 1.20	1.79 ± 0.77
Hentriacontane	0.44 ± 0.26	1.53 ± 0.24	nd	1.12 ± 1.03	nd	1.00 ± 0.59
Total	5.99	15.56	3.70	6.89	13.91	10.74

Data is means ± SD of five replicates (sub-locations).

*Indicates statistically significant differences between locations means (P < 0.05).

### Correlation analysis between bilberry wax composition and climatic variables

**FIGURE 2:**
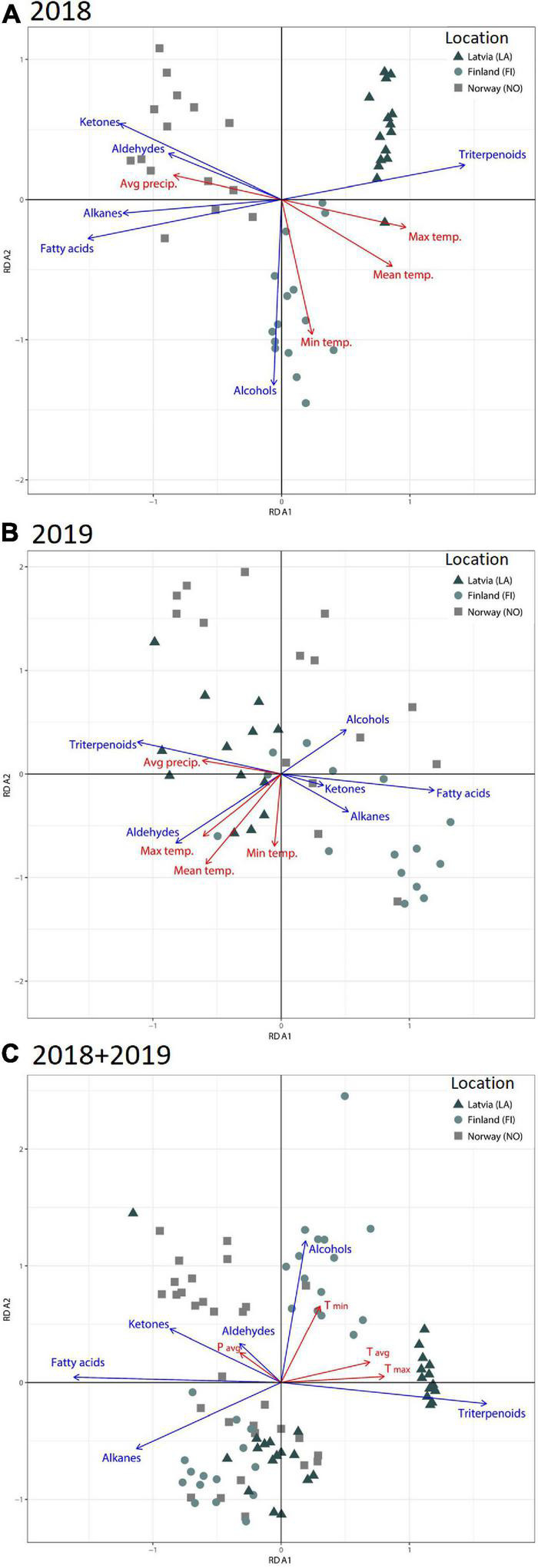
RDA analysis presenting groups of compounds of berry wax and weather variables in different latitudinal locations in years **(A)** 2018, **(B)** 2019, and **(C)** combined data from 2018 and 2019.

Redundancy analysis (RDA) was used to analyze the variation in cuticular wax composition of berries from different latitudes. The analysis clearly distinguished between the bilberries growing in three separate locations harvested in 2018 (Figure 2A). Berries collected in 2019 showed larger variance and the different locations formed overlapping clusters (Figure 2B). Total explained variation by the RDA from the season 2018 was 74.2 and 45.2% for the 2019 data (Figures 2A,B). The total variance for the combined data for RDA analysis was 64.7% (RDA1 48.2%; RDA2 16.5%) (Figure 2C). The calculated RDA1 and RDA2 are significant for all the analyzed data sets (2018; 2019; 2018 and 2019 combined, *p* < 0.001).

**FIGURE 3:**
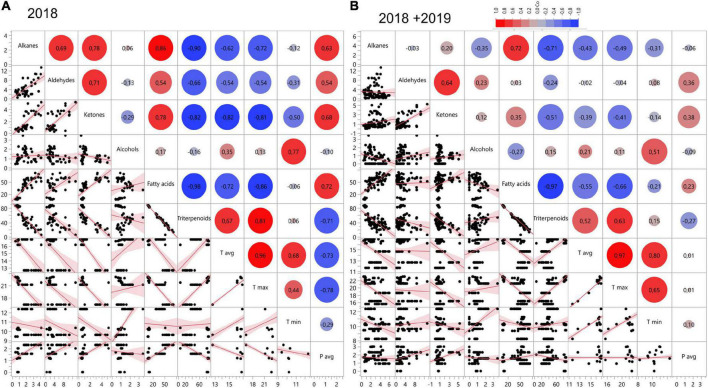
Correlation matrix showing relationship between wax compounds and climatic factors averaged through 8 weeks pre-harvest in **(A)** 2018 and **(B)** 2018 and 2019. *X* and *Y* axis in the correlation matrix can represent either the relative amount of each of the respective groups of compounds, ^°^C in the case of temperature (T_avg_, T_max_, T_min_) or mm for precipitation (P_avg_).

Redundancy analysis showed negative associations between triterpenoid and fatty acid content in the cuticular wax (Figures 3A,B). In 2018, triterpenoid content indicated a positive correlation with the T_max_ (0.81; *p* < 0.0001) and T_avg_ (0.67; *p* < 0.0001) (Figure 3A). In the year 2018, fatty acids showed significant negative correlation with the T_avg_ (–0.72; *p* < 0.0001) and T_max_ (–0.86; *p* < 0.0001) (Figure 3A). Alkanes, ketones, and aldehydes also showed significant negative correlation with T_avg_ and T_max_ in 2018. The correlation analysis for the combined data from 2 years also showed positive correlation between triterpenoids and T_max_ (0.63; *p* < 0.0001) and T_avg_ (0.52; < 0.0001) (Figure 3B). A negative correlation was seen between fatty acids and T_max_ (–0.66; *p* < 0.0001) and T_avg_ (–0.55; *p* < 0.0001). The overall RDA analysis indicates that temperature affects the composition of cuticular wax, and specifically, the triterpenoid fraction in a positive manner.

A stepwise linear regression analysis was conducted to identify the significant climatic factors and time periods that may have influenced the bilberry fruit cuticular wax composition. The analysis showed that the variation in composition of triterpenoids was highly affected by the climate parameters (*R*^2^ = 88%). The proportion of triterpenoids was positively correlated with T_max_ as well as T_avg_ from the beginning of summer season (Supplementary Table 3). The variation in aldehyde composition was the least affected by location and climate (*R*^2^ = 37.2%), while for the composition of other VLC compounds, alkanes (*R*^2^ = 76.4%), ketones (*R*^2^ = 75.0%), fatty acids (*R*^2^ = 88.7%), and alcohols (*R*^2^ = 81.8) were much more affected by climate and location of growth. Composition of fatty acid correlated negatively with T_max_ and T_avg_ throughout the studied season (Supplementary Table 4).

### Controlled temperature experiment

**FIGURE 4:**
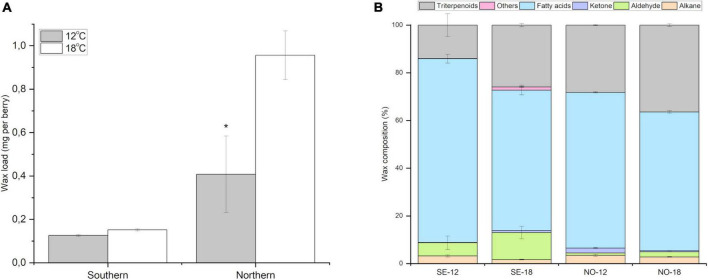
Effect of temperature on berry cuticular wax. **(A)** Amount of cuticular wax (mg per berry) in bilberry fruits after phytotron experiments at 12 and 18°C in southern and northern bilberry clones. The data is shown as mean ± SD of three replicates (*n* = 3). * Indicates the significant difference between the two temperature treatments (*P* < 0.05) by student *t*-test. **(B)** Cuticular wax profile in bilberries from phytotron experiments from southern and northern clones at 12 and 18°C. Error bars indicate Standard Error (*n* = 3).

In order to validate the role of temperature on wax load and composition in more detail, a controlled phytotron experiment was conducted, through which the accumulation and composition of cuticular wax in berries ripening at 12 and 18°C was compared in southern and northern bilberry ecotypes. After 14 days temperature treatment, the highest wax load was detected in the berries of the northern clones ripening at 18°C compared to berries ripened at 12°C or to southern ecotypes in both temperatures (Figure 4A). Northern clones showed 134% increase in berry wax load at 18°C compared with berries ripened at 12°C. Southern clones showed no significant difference in wax load between the two temperatures.

Berries from northern and southern ecotypes showed a similar trend of change in the proportion of wax compounds among berries ripening at 18°C compared to 12°C (Figure 4B). The relative proportion of triterpenoids in berry cuticular wax was found to be higher in bilberries ripening at 18°C compared to 12°C (85% in southern clones, 29% in northern clones). The relative proportion of aldehydes was also higher (104% in southern clones and 107% in northern clones) in berries ripening at 18°C compared to 12°C (Figure 4B). For fatty acids, the relative proportions were found to be lower (30% in southern clones, 12% in northern clones). Similarly for alkanes, the relative proportion wax lower (94% in southern clones, and by 23% in northern clones) in berries at 18°C compared to 12°C.

The profiles of chemical compounds changed differently in the northern and southern clones of bilberry. In the northern clones, friedelin contributed mostly to the increase in triterpenoids proportion in 18°C (18.3%) compared to 12°C (4.7% of total wax) (Table 3). In the berry cuticular wax of the southern clones, the increase in triterpenoid content was contributed mostly by oleane backbone compound, olean-2.12-dien-28-oic acid (Table 3). Olean-2.12-dien-28-oic acid content was higher in berry fruit cuticular wax in clones grown at 12°C (2.2% of total wax) compared to the ones grown at 18°C (8.7% of total wax). Oleanolic acid was found to exist only in southern clones and in higher proportion at 18°C (7% of total wax) than at 12°C (2% of total wax). In the southern clones, the higher aldehyde proportion was mainly contributed by hexacosanal, octacosanal and nonacosanal, while in northern clones, heptacosanal and nonacosanal contributed to the increasement (Table 3). The decrease in fatty acid content was mainly contributed by eicosanoic acid (14.9% of total wax at 18°C, 6.97% of total wax at 12°C) and lignoceric acid (5.75% of total wax at 18°C, 1.3% at 12°C) in southern clones. In case of alkanes, the decrease was mainly contributed by pentacosane and heptacosane (Table 3).

**TABLE 3:** Quantities (μg/mg of berry cuticular wax) of cuticular wax compounds in ripe bilberry wax in phytotron experiments.

Cuticular wax compounds	Southern clones		Northern clones	
		
	12°C	18°C	12°C	18°C
**Triterpenoids**				
Friedelin	9.07 ± 7.91	nd	48.25 ± 4.78*	150.63 ± 6.58*
Oleanolic acid	11.38 ± 19.91	23.01 ± 0.63	nd	nd
Ursolic acid	nd	nd	nd	nd
β-Amyrin	17.13 ± 11.16	11.33 ± 0.49	91.48 ± 7.21*	17.53 ± 0.87*
α-Amyrin	14.00 ± 3.38	10.22 ± 0.38	19.22 ± 0.28*	69.68 ± 0.41*
Lupeol	16.26 ± 4.47	12.28 ± 0.42	nd	nd
Olean-2.12-dien-28-oic acid 3-one	12.29 ± 21.29	28.62 ± 0.77	28.95 ± 1.47*	12.63 ± 2.18*
Ursa-2.12-dien-28-oic acid	nd	nd	24.77 ± 1.69	19.40 ± 0.57
**Fatty acids**				
Eicosanoic acid	82.21 ± 10.21*	22.99 ± 2.37*	108.20 ± 20.32	119.83 ± 8.61
Lignoceric acid	4.30 ± 0.32*	31.39 ± 4.98*	54.22 ± 1.19*	36.10 ± 1.07*
Cerotic acid	56.92 ± 1.14*	126.48 ± 33.07*	209.93 ± 15.29*	117.37 ± 5.18*
Montanic acid	91.23 ± 3.19*	131.04 ± 15.38*	107.86 ± 3.36	110.82 ± 1.18
**Alkanes**				
Pentacosane	4.03 ± 0.55*	0.61 ± 0.07*	2.83 ± 2.80	5.64 ± 0.37
Hexacosane	0.82 ± 0.74	0.34 ± 0.01	1.52 ± 1.01	1.20 ± 0.11
Heptacosane	8.83 ± 0.92*	1.92 ± 0.24*	11.38 ± 0.99	10.23 ± 1.43
**Aldehydes**				
Pentacosanal	0.81 ± 0.98	0.22 ± 0.07	nd	nd
Hexacosanal	2.32 ± 1.55*	9.98 ± 3.15*	1.71 ± 0.68	0.73 ± 0.15
Heptacosanal	6.34 ± 8.58	0.97 ± 0.35	1.20 ± 0.27	1.38 ± 0.08
Octacosanal	11.38 ± 9.55	15.63 ± 4.70	3.92 ± 0.73	2.46 ± 0.18
Nonacosanal	nd	9.77 ± 16.05	1.31 ± 0.30*	2.86 ± 0.21*
Triacontanal	6.46 ± 5.75	0.96 ± 0.33	nd	3.05 ± 0.08
Hentriacontanal	0.33 ± 0.30	nd	nd	nd

Data is means ± SD of three replicates.

*Indicates statistically significant differences between means at 12 and 18°C (P < 0.05).

## Discussion

### Temperature has a major role in modifying bilberry fruit cuticular wax composition

Most studies investigating the effects of temperature on cuticular wax composition of plants have focused on *n*-alkanes due to their role as plant biomarkers (Dodd and Poveda, 2003; Wu M. S. et al., 2017; Wang et al., 2018), while other compounds have been less studied. Previous cuticular wax studies in fruits have focused to study the temperature effects during post-harvest storage. Our results present the first comprehensive report on the effect of temperature on all wax components on fruit, through a latitudinal gradient as well as controlled phytotron study with southern and northern bilberry ecotypes.

Based on our results, including RDA analysis, temperature was found to have a major effect on the compositional changes of bilberry fruit waxes through the latitudinal gradient. We observed a trend of decrease in triterpenoid content and proportions along with increase in fatty acids, aldehydes, and alkanes in bilberry fruit cuticular wax through the latitudinal gradient from Latvia through Finland to northern Norway in the two studied seasons. The results from the cuticular wax samples of the northernmost latitude in 2019 did not follow this trend. Several other factors, such as fluctuation in light radiation levels, photoperiod, and humidity, may also interplay in determination of the cuticular wax composition in natural environment (Shepherd and Griffiths, 2006; Lihavainen et al., 2017). Also, the mean daily radiation and the light conditions vary in different latitudes (Johansen et al., 2017; Mølmann et al., 2021). The daylength in July increases from 15–17 h in the southern growth location to up to 22–24 h in the northern most location of our study. It has been reported that cuticular wax load and composition is affected by light and dark cycles for several plant species (Giese, 1975; Shepherd et al., 1995; Go et al., 2014). Besides day length, the mean daily radiation is also influenced by cloudiness, which may have affected the detected differences in wax formation between the different seasons. It is known that the relationship between wax composition and natural environmental gradient is confounded by complex interactions among various environmental factors as well as by genetic effects (Dodd and Poveda, 2003; Gosney et al., 2016).

To disentangle temperature from other effects and verify the role of temperature in variation in chemical composition of bilberry fruit cuticular wax, we conducted controlled temperature experiment in phytotrons. The results confirmed the effect of temperature on the compositional trends of bilberry cuticular wax. In the controlled phytotron study, a marked increase in the content and proportion of triterpenoids with the increase in temperature was observed in bilberry fruit cuticular wax in both southern and northern clones. Interestingly, different compounds contributed to the higher proportion of triterpenoids at higher temperature in northern and southern clones of bilberry. Oleane backbone compounds reacted more to higher temperature in southern clones, while in northern clones friedelin accumulation was changed. These results signify that both the genetic factors and the climatic variables modify the berry cuticular wax composition.

It has been reported that the content of plant volatile monoterpenes generally increases with warmer temperatures, but terpenes with higher number of isoprene units are not as responsive to temperature changes similarly to other secondary metabolites (Holopainen et al., 2013). However, our study showed a clear increase in triterpenoid content in bilberry fruit cuticular wax with increase in temperature. Similarly, after post-harvest cold storage (0°C), an increase in proportion of triterpenes in sweet cherry fruit has been reported (Belge et al., 2014). Therefore, it seems that triterpenoids in cuticular wax of fruits are responsive to temperature changes. The functional role of higher content of triterpenoids with increase in temperature could be related to protection of the berry against thermal stress. It has been proposed that triterpenoids in cuticular wax play a role in restricting thermal expansion of the cuticle, hence, protecting it against thermal damage (Schuster et al., 2016). Therefore, we suggest that with predicted global temperature increase due to climate change, triterpenoid content in bilberry cuticular waxes may increase to prevent thermal stress. This phenomenon needs further studies in other species and organs as well.

Among very long chain fatty acid (VLCFA) compounds, we observed a decrease in the proportion of alkanes and fatty acids with the increase in temperature in our phytotron study. Some studies conducted in cold storage temperatures (0–4°C), have reported that an increase in proportion of fatty acids and alkanes occurs under lower temperature conditions. For example, an increase in the content of alkanes and secondary alcohols was reported in the cuticular wax of *Arabidopsis*, when seedlings were grown at cold temperature (4°C) temperature as compared to the ones grown at 23°C (Ni et al., 2014). Similarly, an increase in the proportion of fatty acids was observed after exposure to cold stress in *Thellungiella salsuginea* leaves (He et al., 2019). Our study aligns with the conclusion that an increase in temperature can lead to the decrease in fatty acids and alkanes in cuticular wax and similarly, a decrease in temperature leads to increase in fatty acids and alkanes in cuticular wax. The role of fatty acids in cuticular wax is to maintain the impermeable cuticle barrier, and it has been shown that fatty acids, especially unsaturated ones, play a major role in protecting plant tissues during cold stress (Yadav, 2010; He et al., 2019).

### Profiles of very long chain fatty acid and triterpenoids show different trends through the latitudinal gradient

The analysis of triterpenoid and VLC aliphatic compounds profile indicated that the dominant component of triterpenoid fraction changes through the latitudinal gradient, while it remains the same for VLC aliphatic compounds. In berries from the southernmost latitude (Latvia), oleanolic acid (triterpene acid) was the dominant compound, while in more northern latitudes (Finland and Norway), β-amyrin (triterpene alcohol) dominated the triterpenoid composition in both seasons. Overall, oleanolic acid and β-amyrin were the main compounds contributing to proportion change through the latitudinal gradient. Our results suggest that the switch between β-amyrin and oleanolic acid could be majorly caused due to the variation in environmental conditions. In our previous study (Trivedi et al., 2019a), where wax samples were collected in Oulu, Finland in 2017, β-amyrin was the dominant triterpenoid, while in this study, although berries were collected from the same locations in 2018 (Trivedi et al., 2021), oleanolic acid was the dominant triterpenoid. Therefore, it seems that the dominant triterpenoid compound changes, especially oleanolic acid levels, are more affected by the changing environmental factors and less by the genetic factors.

In contrast to triterpenoids, montanic acid (fatty acids), heptacosane (alkanes), and 2-heneicosanone (ketones) were the dominant components in all the studied geographical locations with the changing climatic conditions. Therefore, the dominant components of fatty acids, alkanes and ketones appeared not to respond to climate factors, but the carbon chain lengths of dominant compounds are more fixed characteristics linked mostly to the genetic differences between bilberry populations. A similar result for alkanes concluded that *n*-alkane characteristics and chain length did not vary with climate variability (Andrae et al., 2019). Based on our study, the VLC pathway and the chain lengths of its derivatives are precisely controlled in bilberry fruit cuticular wax, thus, the dominant compounds of all VLC derivatives remain same through the geographical locations and latitudinal gradient. The biosynthetic route to triterpenoid components on the other hand seems to be more sensitive to changes in environmental factors.

### Temperature affects bilberry fruit wax load

Cuticular wax load has been shown to be affected by temperature in different ways. A higher wax load was reported at optimal temperature (15°C) as compared to high temperature (35°C) in *Brassica* species (Baker, 1974; Shepherd and Griffiths, 2006). Recent studies have shown an increase in the total wax load in the cuticular wax of Arabidopsis plants and *Thellungiella salsuginea* leaves, after exposure to cold stress (Ni et al., 2014; He et al., 2019). However, some post-harvest studies in fruits have reported a decrease in wax load at cold storage conditions in fruits of blueberry (Chu et al., 2018), Asian pear (Wu X. et al., 2017), sweet cherry (Belge et al., 2014), and apple (Dong et al., 2012). Therefore, the effect of temperature on wax load remains unclear. Our results also showed negative correlation between the wax load and the precipitation (Supplementary Table 3), which is consistent with earlier studies showing negative correlation between higher humidity and wax load in different plant species (Koch et al., 2006; Trivedi et al., 2019b; Wang et al., 2021).

In our phytotron experiment, an increased wax load was observed in berries ripening at higher temperature (18°C) as compared to the ones at 12°C. Interestingly, we observed that the increase in wax load at higher temperature was more pronounced in northern than southern clones of bilberry. Northern clones of bilberry were more sensitive to temperature increase in terms of cuticular wax load alterations probably due to adaptation to lower temperatures. Overall, our results indicate that an increase of few degrees in average temperature, will potentially lead some bilberry populations to markedly increase their wax load. However, if the temperature higher than 18°C would have shown the same effect in southern bilberry clones, remains to be investigated.

## Data availability statement

The original contributions presented in this study are included in the article/Supplementary material, further inquiries can be directed to the corresponding author.

## Author contributions

LK, LJ, HH, MK, IM, AH, KK, and PT conceived the idea and planned the experiments. PT, LK, and AH collected the samples. PT and LK performed the wax extractions. LK and JK performed the GC-MS analysis. AH and DE performed the correlation and statistical analysis. All authors discussed the results, contributed to the manuscript preparation, and approved the final version of the manuscript.
