# Supplementary material for: Temperature has a major effect on the cuticular wax composition of bilberry (Vaccinium myrtillus L.) fruit
Source: Front Plant Sci. 2022 Sep 20;13:980427. doi: 10.3389/fpls.2022.980427 (PMC9530925; doi:10.3389/fpls.2022.980427)
Supplement: Supplementary file 1 [file Data_Sheet_1.pdf]

Supplementary Material

Supplementary Table S1: Coordinates and date of harvest for bilberry fruit collection from 3 geographical location (5 sublocations)

**Latvia**				
	2018		2019	
**Sub-location**	Coordinates (latitude, longitude degree)	Date of harvest	Coordinates	Date of harvest
1.	57.464188, 25.337263	15 Jul	57.464188, 25.337263	14 Jul
2.	56.793159, 23.654256	16 Jul	56.793159, 23.654256	16 Jul
3.	57.174855, 27.121943	14 Jul	57.174855, 27.121943	20 Jul
4.	56.699719, 22.806207	10 Jul	56.699719, 22.806207	14 Jul
5.	57.134889, 21.828712	16 Jul	57.134889, 21.828712	16 Jul
**Finland**				
	2018		2019	
	Coordinates (latitude, longitude degree)	Date of harvest	Coordinates	Date of harvest
1.	65.058444, 25.460472	31 Jul	65.058444, 25.460472	12 Aug
2.	65.074194, 25.446167	31 Jul	65.074194, 25.446167	12 Aug
3.	65.190265, 25.341861	1 Aug	65.190265, 25.341861	12 Aug
4.	65.078250, 25.530694	1 Aug	65.078250, 25.530694	12 Aug
5.	65.068917, 25.527861	1 Aug	65.068917, 25.527861	12 Aug
**Norway**				
	2018		2019	
	Coordinates (latitude, longitude degree)	Date of harvest	Coordinates	Date of harvest
1.	69.651889, 18.912333	13 Aug	69.648889, 18.912333	23 Aug
2.	69.685083, 18.972194	13 Aug	69.685083, 18.972194	23 Aug
3.	69.693944, 18.988972	13 Aug	69.693944, 18.988972	23 Aug
4.	69.560000, 18.726111	13 Aug	69.560000, 18.726111	23 Aug
5.	69.533326, 18.394741	13 Aug	69.533326, 18.394741	23 Aug

Supplementary Table S2. Climate data (averaged for 8 weeks growth period prior to harvest) used for correlation analysis.

Coordinates	Location	Mean Temp (°C)	Min Temp (°C)	Max Temp (°C)	Precipitation (mm)
	2018				
69°N 18°E	Norway	12.65	9.61	16.34	2.30
65°N 25°E	Finland	16.93	12.38	21.22	1.55
56°N 24°E	Latvia	16.52	10.08	22.58	1.20
	2019				
69°N 18°E	Norway	11.65	8.23	15.54	1.36
65°N 25°E	Finland	14.68	10.25	18.59	0.91
56°N 24°E	Latvia	16.62	10.62	22.04	2.54

Supplementary Table S3. Pearson correlation of bilberry wax composition data corresponding to monthly climate data at different times periods before harvest (first: third month before harvest, mid: second month before harvest, last; first month before harvest). Increasing correlation coefficient is indicated with increasing color intensity: red (negative) and blue (positive).

Correlation	Temperature (max)			Temperature (avg)			Temperature (min)			Precipitation (avg)		
	First	Mid	Last	First	Mid	Last	First	Mid	Last	First	Mid	Last
Alkanes	-0.339	-0.307	-0.533	-0.278	-0.411	-0.462	0.089	0.049	-0.437	0.212	-0.357	-0.600
Aldehydes	-0.422	-0.309	0.154	-0.458	-0.291	0.185	-0.361	-0.192	0.285	0.045	0.469	0.539
Ketones	-0.751	-0.566	-0.208	-0.725	-0.649	-0.154	-0.321	-0.463	0.003	0.331	0.576	0.462
Alcohols	0.014	-0.440	0.637	0,150	-0.092	0.674	0.372	-0.116	0.798	-0.245	0.614	0.195
Fatty acids	-0.587	-0.517	-0.505	-0.543	-0.577	-0.403	-0.017	0.054	-0.307	0.063	-0.291	-0.410
Triterpenoids	0.639	0.593	0.438	0.584	0.623	0.327	0.027	-0.015	0.209	-0.063	0.174	0.334
Total load	0.282	0.325	-0.235	0.222	0.267	-0.255	0.029	0.313	-0.348	0.305	-0.765	-0.429

Supplementary Table S4. Results from linear mixed-effect model predicting effect of climate (mean, max, min temperature, and average precipitation last 8 weeks before harvest) and location on the bilberry wax load.

Mixed-effect model (8 weeks preharvest)	
Tests of Fixed Effects	*P*-value
Avg temperature	0.500
Max temperature	0.134
Min temperature	0.510
Avg precipitation	0.569
Location	0.000
